# Cost-Sensitive Learning for Anomaly Detection in Imbalanced ECG Data Using Convolutional Neural Networks

**DOI:** 10.3390/s22114075

**Published:** 2022-05-27

**Authors:** Muhammad Zubair, Changwoo Yoon

**Affiliations:** Electronics and Telecommunication Research Institute, Daejeon 34129, Korea; cwyoon@etri.re.kr

**Keywords:** arrhythmia detection, ECG classification, cost-sensitive learning, imbalanced data, convolutional neural networks

## Abstract

Arrhythmia detection algorithms based on deep learning are attracting considerable interest due to their vital role in the diagnosis of cardiac abnormalities. Despite this interest, deep feature representation for ECG is still challenging and intriguing due to the inter-patient variability of the ECG’s morphological characteristics. The aim of this study was to learn a balanced deep feature representation that incorporates both the short-term and long-term morphological characteristics of ECG beats. For efficient feature extraction, we designed a temporal transition module that uses convolutional layers with different kernel sizes to capture a wide range of morphological patterns. Imbalanced data are a key issue in developing an efficient and generalized model for arrhythmia detection as they cause over-fitting to minority class samples (abnormal beats) of primary interest. To mitigate the imbalanced data issue, we proposed a novel, cost-sensitive loss function that ensures a balanced deep representation of class samples by assigning effective weights to each class. The cost-sensitive loss function dynamically alters class weights for every batch based on class distribution and model performance. The proposed method acquired an overall accuracy of 99.81% for intra-patient classification and 96.36% for the inter-patient classification of heartbeats. The experimental results reveal that the proposed approach learned a balanced representation of ECG beats by mitigating the issue of imbalanced data and achieved an improved classification performance as compared to other studies.

## 1. Introduction

Electrocardiogram (ECG) is a non-invasive diagnostic tool that is widely used to monitor the function of the heart over time. It represents the electrical activity of the heart and plays an important role in identifying various cardiovascular diseases such as arrhythmia, myocardial infarction, and Ventricular Tachycardia [1]. Among various cardiovascular abnormalities, arrhythmia is the most common heart disease that results from disturbance in the rate, rhythm, or conduction of electrical signals through the heart [2]. Cardiac abnormality associated with different types of arrhythmia provokes various types of beat patterns and can thus be detected by analyzing ECG waveforms. However, it is difficult for a cardiologist to detect arrhythmia from ECG signals recorded over a short period of time as arrhythmia may appear suddenly and infrequently. Therefore, long-term ECG recording is needed in order to successfully capture these infrequently occurring abnormal beats. For long-term recordings, a Holter device is used, which can record ECG signals for 24 h or longer. Such long ECG recordings contain hundreds of thousands of beats, which makes it difficult for a cardiologist to visually interpret each beat. Moreover, the visual and manual investigation of long-term ECG data is a tedious job and prone to human error. Therefore, the automatic classification of heartbeats is of utmost importance to mitigate the issues related to the visual interpretation of long-term ECG recordings.

The automatic classification of ECG beats is a challenging task, and regardless of the efforts made by researchers, it is still considered an open problem due to several reasons. For instance, the morphological characteristics of ECG waveform varies from patient to patient over time, depending on the existing physiological processes and mental state of the patients [3]. The physiological processes and the activity of the autonomic nervous system may influence the intervals (RR interval, QRS interval) and segments (PR segment, ST segment) of ECG waveforms [4]. Therefore, the arrhythmia detection model designed with hand-crafted features for a specific group of patients may not perform well for other patients. Thus, the over-reliance on hand-crafted features should be avoided in order to achieve better generalizability for the classification model. Similarly, the failure to adopt a common procedure for the training and evaluation of heartbeat classification models is also a major issue. The recommendation provided by the Association for the Advancement of Medical Instrumentation (AAMI) should be adopted while developing a heartbeat classification model [5,6]. According to the recommendation of AAMI, all types of ECG beats can be categorized into five groups: N (normal beats), S (supraventricular ectopic beats), V (ventricular ectopic beats), F (fusion beats), and Q (unclassifiable beats). Table 1 illustrates the categorization of ECG beats based on AAMI standards.

Another crucial problem is the class imbalance of data, in which the training data have a skewed distribution of class instances. In imbalanced data, some classes (minority classes) have scarce representation while other classes are highly abundant. During training, the high representation of the majority class forces the classification algorithm to be biased towards the majority class. As a result, the minority class samples are classified as majority class samples [7]. However, in certain real-world scenarios, less frequently occurring events are of great importance. For instance, suspicious activity in a surveillance task is a rare event that should be recognized correctly by the monitoring system [8]. Similarly, in medical applications, disease diagnosis tasks are examples of such scenarios where minority class samples are of particular interest [9]. One such scenario is the detection of arrhythmia since it appears suddenly and infrequently [10]. In such cases, the detection of abnormal beats is of utmost importance, and the erroneous classification of abnormal beats as normal is highly undesirable. Therefore, it is crucial for a heartbeat classification algorithm to handle the problem of imbalanced data in order to have a higher identification rate for abnormal heartbeats.

In this paper, we propose a deep learning-based beat classification approach to mitigate the aforementioned challenges in heartbeat classification. The proposed approach can efficiently classify different heartbeats without using any of the hand-crafted ECG features. Moreover, the proposed model is based on the recommendations provided by AAMI. The contribution of this paper can be summarized as follows:

- We developed a novel deep learning model that can efficiently learn the deep representation of heartbeats by extracting different temporal information using temporal transition modules with multiple kernels of variable depths. The transition module is used to capture short-term and long-term variations of ECG waveforms for an effective deep representation learning.
- To improve the classification rate of minority classes, we designed a new cost-sensitive loss function that can efficiently mitigate the problem of imbalanced data in convolution neural networks.

The rest of the paper is organized as follows. Section 2 provides a survey of related literature with explanations of the basic concepts of ECG beat classification. Heartbeat classification methodology, including data description, pre-processing, segmentation, and illustration of the proposed deep model, and a cost-sensitive strategy are presented in Section 3. The classification results of the proposed method and a performance comparison are reported in Section 4. The concluding remarks are presented in Section 5.

## 2. Related Work

In the last decade, arrhythmia detection has been widely investigated, and numerous studies suggesting various approaches for abnormal heartbeat identification have been reported. Many of these studies focused on traditional approaches that are commonly based on some basic sequential steps [11]. First, pre-processing is performed to eliminate different noises (motion artefacts, power line interference, etc.) from ECG recordings. After noise removal, various fiducial points (P wave, R peak, QRS complex, T wave) are identified and extracted in order to capture the morphological information of the heartbeat. There is a considerable amount of literature available on pre-processing [12] and fiducial point extraction [13,14,15]. The second step includes feature extraction from ECG segments. Many of the previously reported ECG beat classification algorithms are based on hand-crafted features extraction techniques such as wavelet transform [16], higher-order statistics [17], Hermit function [18], morphological features [2], etc. Finally, the extracted features are classified using different machine learning algorithms such as support vector machine [19], decision trees [20], artificial neural networks [21,22], etc.

These machine learning methods adopt conventional signal processing techniques to extract morphological characteristics from ECG signals. However, the morphological patterns of ECG beats vary from subject to subject, which causes variability in the hand-crafted features of different subjects. Similarly, under different circumstances, the environmental stimuli modulating ECG waveforms cause variations in the morphological characteristics, which leads to inconsistent results. Therefore, these methods are not suitable for use in Arrhythmia detection in a larger population.

To overcome the issues of hand-crafted ECG features, many approaches based on deep learning have been proposed for heartbeat classification. The primary objective of these studies was to improve model generalization while negating the need for hand-crafted features. These studies can be categorized into two groups according to their experimental paradigm: intra-patient paradigm and inter-patient paradigm. In an intra-patient classification of ECG beats, each patient’s data are divided into training and test sets. In other words, each patient’s data are used for both training and evaluation. The majority of the studies on beat classification adopts the intra-patient paradigm. For instance, Acharya et al. [23] developed a deep convolutional neural network (CNN) for five-class beat classification. ECG segments with 260 samples centered around the R-peak were used as inputs. In this study, for model training and evaluation, the authors employed the k-fold cross-validation approach. CNN-based models for heartbeat classification were also proposed in [24,25,26,27,28,29]. In addition, generative adversarial networks (GAN) have also been employed for intra-patient beat classification [30]. Moreover, long short-term memory networks (LSTM) also demonstrated good results in discriminating different classes of beats. For instance, [1,31] developed arrhythmia detection models based on LSTM and reported good recognition performance for five different beat classes, with a significant reduction in computational cost. Furthermore, a hybrid model of CNN and LSTM was proposed in [32] for arrhythmia detection. The primary goal achieved in this study was the utilization of variable length ECG segments for arrhythmia detection.

The second experimental paradigm is the inter-patient beat classification, which uses different patients’ data for the training and evaluation of the model. These methods mainly focus on the generalization of the model in order to overcome real-world problems and can thus be used for a large population. For example, De chazel et al. [33] trained a model using hand-crafted features extracted from 22 patients’ data. For the evaluation of the model, a separate dataset of 22 patients was used. The performance of beat classification methods based on the inter-patient paradigm truly depicts the generalization of the model and its applicability for a large population. Similar studies based on inter-patient beat classification using a convolutional neural network, a support vector machine, dual fully connected neural networks, and deep belief networks were also presented in [34,35,36,37], respectively.

A challenging issue in developing an arrhythmia detection system is the unequal distribution of beat classes. However, a few researchers have addressed the problem of imbalanced data in arrhythmia detection. For example, a multi-module neural network system was proposed in [38] to overcome the problem of data imbalance in ECG beat classification. For oversampling, borderline-SMOTE algorithms were employed, while a novel context feature module was introduced for feature extraction and selection. These complementary modules aimed to create a balanced set of class samples in order to avoid over-fitting. In another study [39], the authors employed a feature-level fusion technique followed by random oversampling to obtain a balance dataset. The focal loss function, first introduced by Lin et al. [40], addresses the imbalanced data issue during training. Romdhane et al. [41] employed focal loss to develop a heartbeat classification system. The aforementioned studies on imbalanced heartbeat classification significantly mitigate the issues of over-fitting minority classes. However, these studies do not follow the AAMI recommendation for heart beat categorization and model evaluation. In this regard, the most relevant study was presented by Sellami et al. [34]. A batch-weighted loss function was proposed to address the imbalanced beat classification problem using a convolutional neural network. In addition, the beat categorization (five classes) and model evaluation (inter-patient) were performed in accordance with the AAMI recommendations.

Most studies on ECG beat classification tended to focus on intra-patient classification; however, such models may not be used in the real world for a larger population. In addition, the AAMI recommendations for heartbeat categorization and model evaluation have not been followed properly. Furthermore, a key limitation of the previous studies on beat classification is the negligence of the imbalanced data issue. Therefore, we introduce a deep model for ECG beat classification and propose a cost-sensitive learning approach to overcome the issues of over-fitting associated with imbalanced data.

## 3. Methodology

**Figure 1:**
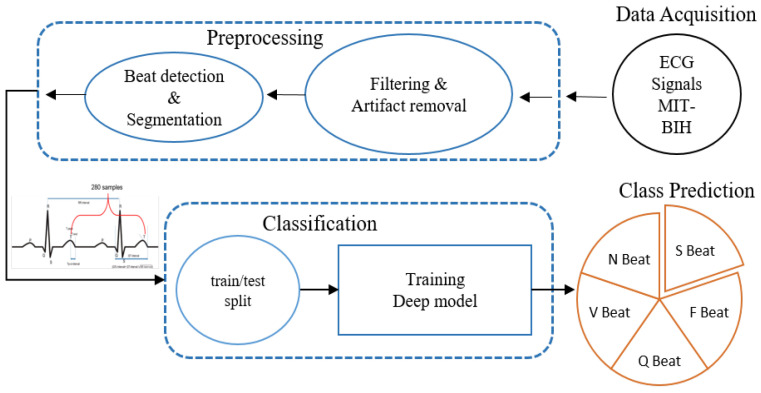
Overview of the heartbeat classification methodology.

The proposed methodology for the deep learning-based heartbeat classification system can be divided into three basic sequential steps, namely data acquisition, pre-processing, and classification. A block diagram of the proposed methodology is given in Figure 1. The first step involves the selection of data from the ECG database for the training and evaluation of the proposed heartbeat classification model. The second step includes the removal of different types of noises and the extraction of beat segments. The third step includes an illustration of the proposed deep classification model and cost-sensitive learning strategy. A detailed description of these steps is given below.

### 3.1. ECG Dataset

In this study, we used the MIT-BIH arrhythmia database [42] for the evaluation of our proposed beat detection approach. This database includes half-hour-long, two-channel ECG recordings of 48 patients. Modified limb lead II signals were used in this study. We selected 44 out of 48 recordings for our experiment and excluded four recordings (102, 104, 107, 217) according to the recommendations of AAMI as these recordings were of low quality and included paced beats.

### 3.2. Pre-Processing and Segmentation

**Figure 2:**
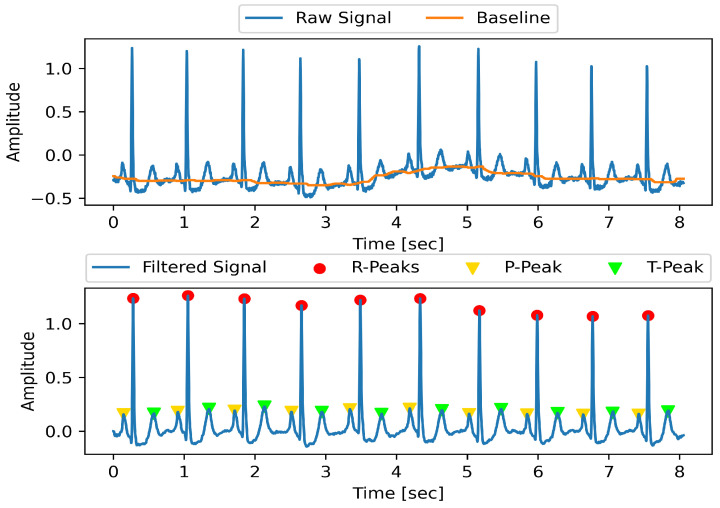
Pre-processing of ECG.

Numerous types of noises deteriorate the quality of ECG signals, which then leads to the poor performance of the classification model. The noises include motion artefacts and power line interference. In this study, we used the same signal denoising technique mentioned by [33]. First, we applied a median filter with a sliding window of 200 ms to remove the P wave and QRS complex from the signal. Then, we applied a second median filter using a 600 ms window to remove the T wave. The output of the second filter contained the baseline of the raw signals. Baseline wander was eliminated by subtracting the second filter output from the raw ECG signal. Furthermore, the signal was passed through a low-pass FIR filter of order n=12. The cut-off frequency of the filter was set at 35 Hz. As a result, power line interference along with high-frequency noises were removed Figure 2.

After filtering all the selected recordings from the MIT-BIH database, we performed ECG peak detection in order to extract the desired segment from the ECG signal. For this purpose, we used a peak detection technique based on the famous Pan and Tompkins algorithm [13]. Extraction of the T wave was also performed. To extract an ECG segment for analysis in the deep model, we extracted two T-to-T segments. The target T segment along with the preceding T segment were extracted. Each segment was re-scaled to 200 samples. After that, both segments were concatenated in order to form a 400-sample-long segment. From all 44 recordings, a total of 100,569 segments were extracted. A summary of the sample class distribution is given in Table 2.

### 3.3. Classification

#### 3.3.1. Model Architecture

The recent literature on heart beat classification reported that convolutional neural networks (CNN) outperformed the conventional methods based on signal processing and machine learning algorithms. These networks have been investigated for a wide area of applications. Among them, 1D convolution has also been investigated for temporal data to capture data patterns. Convolutional neural networks are comprised of a feature extractor and a feature classification part. The feature extractor part learns the high-level feature representation of the input data through stacked convolutional and polling layers, while the classification part classifies these learned features using a fully connected multilayer perceptron. The operation of the convolutional layer can be expressed mathematically as follows:

(1) $$C_{i}^{l,j}=\sigma \left(b_{j}+\sum_{m=1}^{M}w_{m}^{j}x_{i+m-1}^{oj}\right)$$

where σ is the activation function responsible for inducing nonlinearity, *b* is the bias term and *w* is the weight matrix. Similarly the pooling operation (max-pooling) can be given as

(2) $$P_{i}^{l,j}=max_{r\in R}\left(c_{ixT+r}^{l,j}\right)$$

where *R* represents the pooling size and *T* represents the stride rate. The pooling operation reduces the size of the feature map and reduces the influence of distortion. During training, the input data propagate in the forward direction through multiple convolutional and pooling layers, and the feature maps are computed using Equations (1) and (2). At the output, the loss is computed with the help of a cost function, which is then propagated back in order to update the weights accordingly. This process is repeated until the stopping criteria is met.

**Figure 3:**
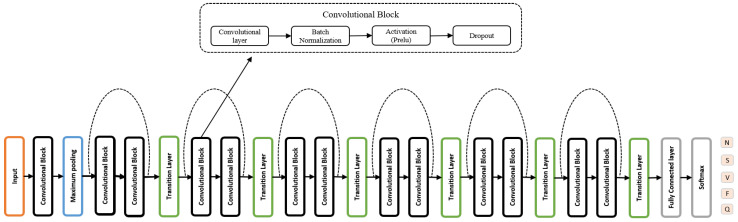
Architecture of the proposed deep learning model.

Figure 3 depicts the architecture of the proposed deep model for arrhythmia detection. It is comprised of a 1D convolutional block and temporal transition modules. Temporal transition modules were used at specific locations after the convolutional block. Each convolutional block includes a convolution layer, followed by a batch normalization, activation, and dropout layer. Batch normalization was used to reduce the internal covariate shift. To overcome the issue of over-fitting, a dropout module was used with a maximum dropout rate of 0.75. After the first convolutional layer, maximum pooling was performed in order to reduce segment dimensionality and computation cost. In the end, a fully connected layer was used, followed by a softmax layer.

#### 3.3.2. Temporal Transition Layer

In the deep learning model, the use of fixed-size kernels homogeneously throughout the network may result in the loss of critical information. Therefore, in this study, we used a temporal transition module to capture the morphological information of the beat. ECG beats exhibit long-term and short-term variations to characterize different beat classes. An analysis of these variations is crucial for the efficient extraction of morphological patterns. The temporal transition module captures the long-term and short-term dynamics of ECG beats with the help of different kernels.

**Figure 4:**
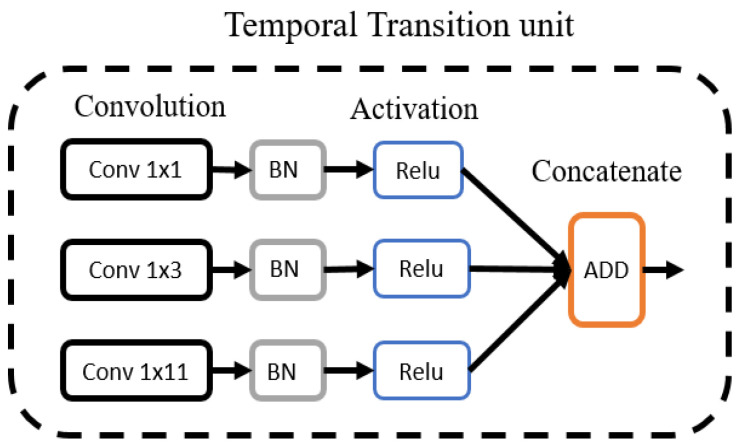
Temporal transition module.

Figure 4 presents the architecture of the temporal transition layer. It includes three convolutional layers with different kernels, followed by the batch normalization and activation layers. For the first two layers, we used kernels with the size of 1 × 1 and 1 × 3 as these kernels aim to extract short-term morphological information. For the third layer, we adopted different kernel sizes (1 × 11, 1 × 15, 1 × 21) during optimization and selected 1 × 11 based on its performance. The feature maps acquired from three convolution layers were concatenated along the filter’s axis. The use of different size kernels assured the extraction of various patterns at the same level in the model, thus preventing the loss of target-specific information with the depth of the model.

#### 3.3.3. Cost-Sensitive Learning

Conventional data-level methods for overcoming class imbalance aim to balance distribution with the use of oversampling. However, the oversampling of minority class samples to create a balanced dataset may cause over-fitting. Therefore, in this study, we introduced a novel, cost-sensitive learning method to mitigate the issue of imbalanced data. Cost-sensitive learning is an algorithm level method used to efficiently train a model with imbalanced data without changing the data distribution [43,44].

The MIT-BIH arrhythmia database is extremely imbalanced, as depicted in Table 2. In such a scenario, the minority classes are less represented, while the majority class gets more representation during training. Therefore, the trained model is more inclined towards the majority class. In other words, the samples coming from the minority classes are classified as majority class samples by the model. The proposed cost-sensitive loss function intelligently assigns different misclassification costs to different classes. It assigns a high misclassification cost to minority class samples, while it penalizes the cost of the majority class samples.

Let us consider an input batch of *M* heartbeats sampled randomly from an imbalanced data set $D={\left\{x_{i},y_{i}\right\}}_{i=1}^{N}$, where *x* represents a beat segment and *y* is the beat class label. Suppose that there are *C* different classes, where C∈{N,S,V,F,Q}. The primary goal is to compute an efficient misclassification cost (ϕ) that significantly overcomes the imbalanced data issue during training. It is important to note that the class weights (CW) of a batch play a key role in computing the cost efficiently. However, in case of extreme imbalance, the class probabilities in a single batch (*M*) sampled randomly may not be equal to the class probabilities of the whole data set (*D*). This difference in distribution significantly deteriorates the performance of the optimizer. Therefore, we compute a quadratic mean to incorporate the impact of both distributions.

(3) $$CW=\sqrt{\frac{{\left(CW_{b}+CW_{D}\right)}^{2}}{2}}$$

where $CW_{b}$ and $CW_{D}$ are class weights of the batch and the whole dataset *D*, respectively. In addition to class weights, numerous performance metrics such as accuracy, sensitivity, and precision are used to adaptively compute the cost during training. In this study, we used the false positive rate (FPR) and the false negative rate (FNR) to assign a significant cost value to each class. These metrics were selected in order to overcome the over-fitting issue caused by imbalanced distribution. To incorporate FPR and FNR into a single quantity, we computed the harmonic mean for the mini-batch as follows:

(4) $$HM_{b}={\left(\frac{{\mathrm{FPR}}^{-1}+{\mathrm{FNR}}^{-1}}{2}\right)}^{-1}$$

Equation (4) gives the harmonic mean of the FPR and FNR. The harmonic mean is considered an appropriate averaging method for rates. In addition, the harmonic mean is not influenced by a higher observation of the FPR or FNR, which makes it suitable for dealing with extremely imbalanced data. We combined the class weights with the harmonic mean to compute the final misclassification cost (ϕ) for the batch. After finalizing the misclassification cost, the total loss was computed, as given below:

(5) $$\phi =CW+HM_{b}$$

(6) $$Loss_{total}=\phi *\left(-\sum_{i}^{c}y_{i}log\left({\hat{y}}_{i}\right)\right)$$

The first term in Equation (6) ϕ is the final misclassification cost, while the second term is the cross-entropy loss for the target mini-batch. The overall loss was computed by multiplying ϕ with the cross-entropy loss in order to get a weighted loss for each class sample.

## 4. Results and Discussion

### 4.1. Experimental Setup

In this study, we proposed a novel deep learning model that can significantly capture small and large-scale temporal and morphological variations with the help of multiple kernels with variable depths. In order to make a fair comparison with other studies, we performed both intra-patient and inter-patient classifications of ECG beats. To evaluate the classification performance of the model under the intra-patient paradigm, we used a k-fold cross-validation approach. In the k-fold cross-validation approach, we split the data into k groups. One group was used for testing, while the remaining K-1 folds of the data were used to train the model. This process was repeated K times, and a new group of data was used as a test set each time. At the end of each trial, different performance measures were obtained. The final results were computed by taking the average of the performance measure acquired in k trials. For the inter-patient classification of beats, we split the data into a training set (DS1) and a test set (DS2), as described in [34]. Both DS1 and DS2 comprised ECG recordings from 22 different subjects. Table 3 summarizes the data distribution of the training set (DS1) and the test set (DS2). For training, we employed the stochastic gradient decent algorithm. In order to handle over-fitting, we used regularization (0.003) in all the convolution layers and dropout (0.75) in the fully connected layers. The initial learning rate was set at 0.01.

### 4.2. Performance Metrics

The classification accuracy treats all classes equally; therefore, a biased model trained with imbalance data can still produce high classification accuracy as it predicts every test sample as a majority class. It does not take into account the misclassification of minority samples, and thus it is not considered appropriate for imbalanced data. For a better evaluation of the proposed method along with the accuracy, we also used sensitivity, specificity, and positive productivity as performance metrics due to their importance in imbalanced learning. Additionally, we also computed the aggregated values of these metrics, as given in [33]. First, we computed the true positive (TP: number of samples correctly indicating association with a specific class), true negative (TN: number of samples correctly indicating disaffiliation with a specific class), false positive (FP: number of samples wrongly indicating association with a specific class), and false negative (FN: number of samples wrongly indicating disaffiliation with a specific class) from the confusion matrix. Then, we computed accuracy, sensitivity, specificity, and positive productivity as follows:

(7) $$\mathrm{Sensitivity}=\frac{\mathrm{TP}}{\mathrm{TP}\;+\mathrm{FN}}$$

(8) $$\mathrm{Specificity}=\frac{\mathrm{TN}}{\mathrm{TN}+\mathrm{FP}}$$

(9) $$\mathrm{Positive}\;\mathrm{Productivity}=\frac{\mathrm{TP}}{\mathrm{TP}+\mathrm{FP}}$$

(10) $$\mathrm{Accuracy}=\frac{\mathrm{TP}+\mathrm{TN}}{\mathrm{TP}+\mathrm{TN}+\mathrm{FP}+\mathrm{FN}}$$

### 4.3. Intra-Patient Classification

In the intra-patient classification of beats, we adopted a 10-fold cross-validation approach. The combined confusion matrix obtained with the 10-fold cross-validation method is given in Table 4. The confusion matrix clearly illustrates the role of cost-sensitive learning in imbalanced heartbeat classification. Despite the extreme imbalanced distribution of the beat classes, the minority class samples (S, V, F, Q) were classified with a lower misclassification rate. The rate of misclassifying the minority classes (S, V, F, Q) into the majority class (N) was significantly low due to the presence of false positive rates (FPR) and false negative rates (FNR). It is important to mention that these two metrics (FPR and FNR) are complementary in cost-sensitive learning. For example, in the absence of FPR, the model will tend to classify minority class samples (S, V, F, Q) into the majority class. Similarly, in the absence of FNR, the excessive emphasis on minority classes will result in the misclassification of the majority class samples.

The performance measures (accuracy, sensitivity, specificity, and positive productivity) are given in Table 5. The proposed method with a cost-sensitive loss function and filters with variable temporal depths achieved 99.81% accuracy, 88.82% sensitivity, 99.54% specificity, and 95.68% positive productivity. The values achieved for sensitivity, specificity, and positive productivity reveal that our proposed loss function efficiently masks the loss function for each class and prevents the model from being biased towards the majority class. Similarly, the stacked convolutional layers and the transition layer efficiently learned the high-level representation of beats by capturing temporal information. The use of the transition layer also assures the extraction of significant features using multi-depth convolutional layers.

### 4.4. Inter-Patient Classification

Under the inter-patient ECG classification paradigm, the data from some patients were used for training, while the remaining data were used for testing. In this way, both the training and test sets included ECG data from different patients. The purpose of this paradigm was to evaluate the model generalization capability so that it could be used for a larger population. We divided the data set into training (DS1) and test sets (DS2) with 22 subjects in each, as proposed by [34]. This experiment of inter-patient classification revealed the generalizability of the system in a true way. Table 6 shows the confusion matrix of the test data (DS2). Although inter-patient beat classification with an extreme imbalanced data distribution is challenging, the proposed method with a temporal transition module and a cost-sensitive loss function proved to be an effective way of improving the generalization and beat discrimination potential of the model.

Additionally, the performance evaluation metrics given in Table 7 clearly depicts the significance of ECG deep representation learning with multiple kernels at the same level. The temporal transition module tends to extract target-specific features while suppressing the subject-specific features, thus improving the generalization of the model. The proposed beat classification method achieved 96.36% accuracy, 70.60% sensitivity, 96.16% specificity, and 48.10% positive productivity for the inter-patient paradigm. These results validate the role of the temporal transition layer in extracting a wide range of morphological features in a generalized manner. However, in comparison to the intra-patient classification of beats, the performance of inter-patient classification is low. This deterioration in performance is caused by inter-subject variations in the morphological characteristics of the ECG signals [3].

### 4.5. Impact of Cost-Sensitive Learning

**Figure 5:**
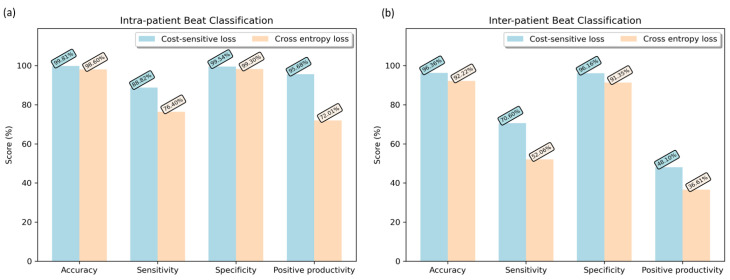
(**a**) Intra-patient beat classification performance comparison between cost-sensitive loss and conventional cross-entropy loss. (**b**) Inter-patient beat classification performance comparison between cost-sensitive loss and conventional cross-entropy loss.

To highlight the impact of cost-sensitive learning on imbalanced beat classification, we trained the same model with a conventional cross-entropy loss function. Figure 5 presents a comparison between two loss functions under the inter-patient and the intra-patient paradigms. The performance metrics (FPR, FNR) with the use of the cost-sensitive loss function significantly overcame the model over-fitting caused by the imbalanced distribution of data. In both experimental paradigms, a substantial increase in all performance metrics was observed. However, the differences in sensitivity (76.40–88.82% for intra-patient and 52.06–70.60% for inter-patient) and positive productivity (72.01–95.68% for intra-patient and 36.61–48.10% for inter-patient) were more prominent as compared to accuracy and specificity. This trend in the performance metrics substantiate the considerable increase in true positive and false negative samples with the use of the cost-sensitive loss function.

### 4.6. Performance Comparison

Table 8 depicts the comparison of our proposed methods’ performance with that of other studies. We evaluated the proposed beat classification method under the inter-patient and intra-patient paradigm. In addition, for a fair comparison on the same basis, only those studies that evaluated their algorithm on the MIT-BIH dataset and followed the AAMI recommendations for beat categorization and model evaluation were considered. Cost-sensitive learning through the proposed loss function significantly increased the classification rate of the minority class samples, as depicted in Table 5. Similarly, in addition to cost-sensitive learning, the transition layers in deep architecture also assured the extraction of high-level features from ECG beats using multiple kernels of variable length. The variable-length kernels facilitated the extraction of short-term as well as long-term variations in the signals. Thus, the morphological features of the P wave, QRS complex, and T wave were deeply analyzed by the model; these features are considered as the key fiducial points of ECG beats and are responsible for the characterization of ECG beats.

The literature on ECG beat classification reveals that the issue of imbalanced data in this domain has been neglected. Unlike [22,24,25,28,29], we addressed the vital issue of imbalanced data in arrhythmia detection and achieved higher classification performance as compared to [22,24,25,28,29]. Acharya et al. [23] used a synthetic ECG sample to overcome the imbalanced data issue; however, generating synthetic samples for minority classes may cause over-fitting. Similarly, in another study [30], a generative adversarial network was employed to augment the minority class samples. In contrast to [23,30], the proposed method in this study mitigated the imbalanced data problem with a novel cost-sensitive loss function and acquired better classification performance. Table 8 also provides a comparison of the proposed method with other studies under the inter-patient paradigm. Under the inter-patient classification paradigm, the beat classification method employing a temporal transition module and a cost-sensitive learning approach also outperformed other studies.

### 4.7. Applications

Deep learning models are widely used in numerous areas for classification tasks. Besides the wide adoption of intelligent models in different areas, the issue of limited and imbalanced data remains everywhere. For instance, in the medical field, the normal data are higher as compared to abnormal data. Thus, in various medical applications such as medical image classification [47] or physiological signals monitoring, there is a need for the handling of the imbalanced data problem in order to better optimize the deep model. Similarly, in machine fault identification, there is a significant difference between normal and erroneous data. For instance, a Point of Sale (POS) system is an important part of the marketplace. Its failure can cause a huge loss and may cause the dissatisfaction of customers. Therefore, fault prediction systems [48] are used to avoid a sudden failure of the system. However, in order to train a prediction model, the available POS data are usually imbalanced and may result in false negative predictions. We believe that the use of a cost-sensitive learning mechanism in such scenarios can lead to a more generalized and accurate deep model.

## 5. Conclusions

In this paper, we proposed a novel algorithm based on deep learning for arrhythmia detection. We introduced a temporal transition module comprised of different convolutional layers with kernels of different sizes. The temporal transition layer was used to capture short-term and long-term morphological variations in ECG beats. We also proposed a cost-sensitive loss function that adaptively assigns special weights to class samples based on model performance and data distribution in the training batch. The proposed method was able to significantly handle the issue of imbalanced data and achieved better classification performance. The experimental results revealed that different convolutional layers with different kernels at the same level (depth) could capture a wide range of target-specific feature maps that improved the classification performance of the model. Similarly, incorporating a false positive rate and a false negative rate of the batch in computing the misclassification cost of the class samples considerably improved the classification rate of the minority class samples. In conclusion, our proposed method can efficiently discriminate between five different types of heartbeats without using hand-crafted features. For future work, we intend to focus on transfer learning in order to handle the issues related to data.

## Figures and Tables

**Table 1:** Mapping of MIT-BIH arrhythmia database beat types to AAMI beat classes.

AAMI Heartbeat Classes Description					
	Normal beat (N)	Supraventricular ectopic beat (S)	Ventricular ectopic beat (V)	Fusion beat (F)	Unknown beat (Q)
	Normal beat (N)	Atrial premature beat (A)	Premature ventricular contraction (V)	Fusion of ventricular and normal beat (F)	Paced beat (/)
	Left bundle branch block beat (L)	Aberrated atrial premature beat (a)	Ventricular escape beat (E)		Fusion of paced and normal beat (f)
**MIT-BIH heart beat types**	Right bundle branch block beat (R)	Nodal (junctional) premature beat (J)			Unclassified beat (Q)
	Atrial escape beat (e)	Supraventricular premature beat (S)			
	Nodal (junctional) escape beat (j)				

**Table 2:** Summary of ECG Beats.

Types	Number of Beats
Normal beats (N)	89,976
Supraventricular ectopic beats (S)	2774
Ventricular ectopic beats (V)	7002
Fusion beats (F)	802
Unknown beats (Q)	15
Total Beats	100,569

**Table 3:** Distribution of training and test sets under the inter-patient paradigm.

Set	Total Subjects	Subject ID	Samples Distribution	
DS1	22	101, 106, 108, 109, 112, 114, 115, 116, 118, 119, 122, 124, 201, 203, 205, 207, 208, 209, 215, 220, 223, 230	N	45,791
			S	939
			V	3785
			F	414
			Q	8
DS2	22	100, 103, 105, 111, 113, 117, 121, 123, 200, 202, 210, 212, 213, 214, 219, 221, 222, 228, 231, 232, 233, 234	N	44,185
			S	1835
			V	3217
			F	388
			Q	7

**Table 4:** Confusion matrix for the intra-patient paradigm.

		Predicted Labels				
		N	S	V	F	Q
True labels	N	89,820	117	34	4	1
	S	122	2636	16	0	0
	V	59	13	6895	35	0
	F	32	3	39	728	0
	Q	1	0	4	1	9

**Table 5:** Classification results for the intra-patient paradigm.

Classes	Accuracy	Sensitivity	Specificity	Positive Productivity
N	99.63%	99.83%	97.98%	99.76 %
S	99.73%	95.03%	99.86%	95.20%
V	99.80%	98.47%	99.90%	98.67%
F	99.89%	90.77%	99.96%	94.79%
Q	99.99%	60.00%	100%	90.00%
Macro	99.81%	88.82%	99.54%	95.68%
Aggregate	99.52%	97.96%	99.83%	98.50%

**Table 6:** Confusion matrix for the inter-patient paradigm.

		Predicted Labels				
		N	S	V	F	Q
True labels	N	40,629	1832	645	989	90
	S	321	1427	83	1	3
	V	224	156	2788	29	20
	F	53	4	50	264	17
	Q	2	0	3	0	2

**Table 7:** Classification results for the inter-patient paradigm.

Dataset	Classes	Accuracy	Sensitivity	Specificity	Positive Productivity
Train Set (DS1)	N	98.47%	98.54%	97.86%	99.76 %
	S	99.26%	93.50%	99.36%	73.41%
	V	99.16%	96.94%	99.34%	92.16%
	F	99.69%	93.24%	99.74%	74.66%
	Q	99.99%	100%	99.99%	66.67%
Macro		99.31%	96.44%	99.26%	81.33%
Aggregate		98.28%	97.82%	98.54%	88.06%
Test Set (DS2)	N	91.63%	91.95%	88.98%	98.54 %
	S	95.16%	77.75%	95.83%	41.74%
	V	97.56%	86.66%	98.32%	78.12%
	F	97.70%	68.04%	97.93%	20.58%
	Q	99.73%	28.57%	99.74%	1.52%
Macro		96.36%	70.60%	96.16%	48.10%
Aggregate		90.89%	88.19%	91.95%	55.75%

**Table 8:** Comparison of classifications for intra-patient and inter-patient beat classification.

Authors	Intra-Patient Beat Classification					
	Classes	Methodology	Acc	Sen	Spe	Ppr
Yun-Chi et al. (2012) [45]	5	clustering	94.30%	93.13%	-	89.50%
Martis et al. (2013) [22]	5	PNN	99.63%	99.83%	97.92%	99.75%
Acharya et al. (2017) [23]	5	CNN+Synthetic data	94.03%	96.71%	91.54%	97.86%
Dang et al. (2017) [24]	5	CNN	95.48%	96.53%	87.74%	-
Raj et al. (2018) [46]	5	SVM	89.93%	72.35%	-	49.29%
Shu Lih Oh et al. (2019) [32]	5	LSTM and CNN	98.10%	97.50%	98.70%	98.69%
Fujita et al. (2019) [28]	4	CNN	98.45%	99.87%	99.27%	-%
Fujita et al. (2019) [29]	4	CNN + CWT	97.78%	99.76%	98.82%	-%
Shu Lih Oh et al. (2019) [25]	5	CNN	98.45%	86.02%	98.40%	87.02%
shaker et al. (2020) [30]	5	GAN	98.30%	99.77%	99.23%	90.00%
**Proposed**	5	CNN	99.81%	88.82%	99.54%	95.68%
Huang et al. (2014) [35]	5	SVM + Threshold	94.55%	96.91%	94.74%	66.68%
Mathews et al. (2018) [37]	5	Deep belief networks	94.13%	66.90%	95.99%	40.98%
A. Sellami et al.(2019) [34]	5	CNN	88.34%	90.90%	88.51%	48.25%
Dechazal et al. (2004) [33]	5	LDA	85.88%	92.85%	86.86%	42.21%
Wang et al. (2020) [36]	4	Dual fully connected neural networks	96.12%	67.68%	95.43%	59.17%
**Proposed**	5	CNN	96.36%	70.60%	96.16%	48.10%

PNN: Probabilistic neural networks, CWT: Continuous wavelet transform, CNN: Convolutional neural networks, GAN: Generative adversarial networks, LDA: Linear discriminant analysis, SVM: Support vector machines.

## Data Availability

The publicly available MIT-BIH Arrhythmia Database can be found here: https://physionet.org/content/mitdb/1.0.0/ (accessed on 24 April 2022).
